# Benzenesulfonohydrazide-tethered non-fused and fused heterocycles as potential anti-mycobacterial agents targeting enoyl acyl carrier protein reductase (InhA) with antibiofilm activity†

**DOI:** 10.1039/d4ra05616g

**Published:** 2024-09-23

**Authors:** Tarfah Al-Warhi, Ahmed Sabt, Małgorzata Korycka-Machala, Asmaa F. Kassem, Moataz A. Shaldam, Hoda Atef Abdelsattar Ibrahim, Malwina Kawka, Bożena Dziadek, Magdalena Kuzioła, Wagdy M. Eldehna, Jarosław Dziadek

**Affiliations:** a Department of Chemistry, College of Science, Princess Nourah bint Abdulrahman University Riyadh Saudi Arabia; b Chemistry of Natural Compounds Department, Pharmaceutical and Drug Industries Research Institute, National Research Centre Dokki Cairo 12622 Egypt; c Laboratory of Genetics and Physiology of Mycobacterium, Institute of Medical Biology of the Polish Academy of Sciences Lodz Poland jdziadek@cbm.pan.pl; d Department of Chemistry, College of Science and Humanities in Al-Kharj, Prince Sattam Bin Abdulaziz University Al-Kharj 11942 Saudi Arabia; e Department of Pharmaceutical Chemistry, Faculty of Pharmacy, Kafrelsheikh University Kafrelsheikh 33516 Egypt wagdy2000@gmail.com; f Pediatric Department, Faculty of Medicine, Cairo University Cairo Egypt; g Department of Molecular Microbiology, Faculty of Biology and Environmental Protection, University of Lodz Lodz Poland; h Bio-Med-Chem Doctoral School of the University of Lodz and Lodz Institutes of the Polish Academy of Sciences Lodz Poland; i Department of Pharmaceutical Chemistry, Faculty of Pharmacy, Pharos University in Alexandria Canal El Mahmoudia St. Alexandria 21648 Egypt

## Abstract

Because resistant variants of the disease are always emerging, tuberculosis is a global issue that affects economies. New antitubercular medications should be developed, and this can be done by inhibiting druggable targets. Enoyl acyl carrier protein (ACP) reductase (InhA) is a crucial enzyme for the survival of *Mycobacterium tuberculosis* (MTB). In this study, a series of small molecules based on non-fused and fused heterocycles (pyridine, coumarin, quinoline, and indole) tethered with benzenesulfonohydrazide were prepared *via* an aza-Michael reaction exploiting a one-pot synthesis approach. The synthesized molecules (2–7) were evaluated for their activity against tubercle bacilli. Three analogues showed efficacy against tuberculosis, with compound 7 demonstrating a MIC value as low as 8 μg mL^−1^. Consequently, compounds 3 and 7 successfully hindered the growth of mycobacteria in human monocyte-derived macrophages (MDMs), demonstrating their ability to penetrate human professional phagocytes. Furthermore, they restricted the ability of mycobacteria to produce biofilms. In addition, the inhibitory effects of compounds 3 and 7 against InhA were assessed. Compound 7 exhibited the best efficacy, with an IC_50_ value of 0.91 μM. The findings showed that the sulfonamide and methyl ester's carbonyl functionalities were engaged in hydrogen bonding with the essential Ile194 and Tyr158 residues, respectively.

## Abstract 2

Because resistant variants of the disease are always emerging, tuberculosis is a global issue that affects economies.

## Graphical abstract

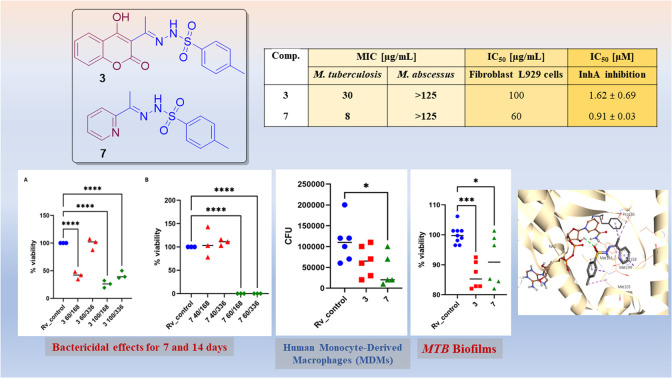

## 1. Introduction

The spread of infectious diseases has long been a significant worldwide health concern because of how quickly germs can be transmitted in various environments. This issue is particularly challenging in densely populated regions, where controlling the spread of diseases from person to person is a significant challenge. While numerous medications are available to treat these infections, a concerning trend is the increasing resistance of microbes to most drugs, resulting in the emergence of multidrug-resistant microorganisms. As a result, there is a pressing need to explore new therapeutic compounds to address this serious issue.^1^

Out of all the infectious diseases, tuberculosis (TB) has been identified as the main cause of death caused by a single infectious agent, surpassing HIV/AIDS in rankings. Tuberculosis (TB), also known as ‘white plaque’, is a potentially serious infectious disease caused by the *Mycobacterium tuberculosis* (MTB) complex, which includes various species such as MTB itself, *M. africanum*, *M. bovis*, *M. caprae*, *M. microti*, *M. pinnipedii*, and *M. canettii*. MTB is the main pathogen that primarily affects the lung (pulmonary TB) as well as other vital organs.^2^ According to the WHO, approximately 2 billion individuals worldwide have a latent MTB infection that can stay dormant for many years. Around 5–10% of those infected with latent MTB eventually develop active TB. In addition, in 2022, treatment was only available to about 2 out of 5 individuals suffering from drug-resistant tuberculosis.^3^ Despite the effectiveness of the first-line anti-TB drugs like isoniazid (INH), rifampicin (RIF), ethambutol (EMB), and pyrazinamide (PZA) currently used to treat TB infection, they do not provide optimum results as TB threat is increasing in the developing world.^4,5^ Therefore, it is essential to develop novel anti-TB medicines that exhibit good tolerance and efficacy against both drug-sensitive and drug-resistant strains of MTB, minimal toxicity, and short treatment duration.^6^

**Scheme 1:**
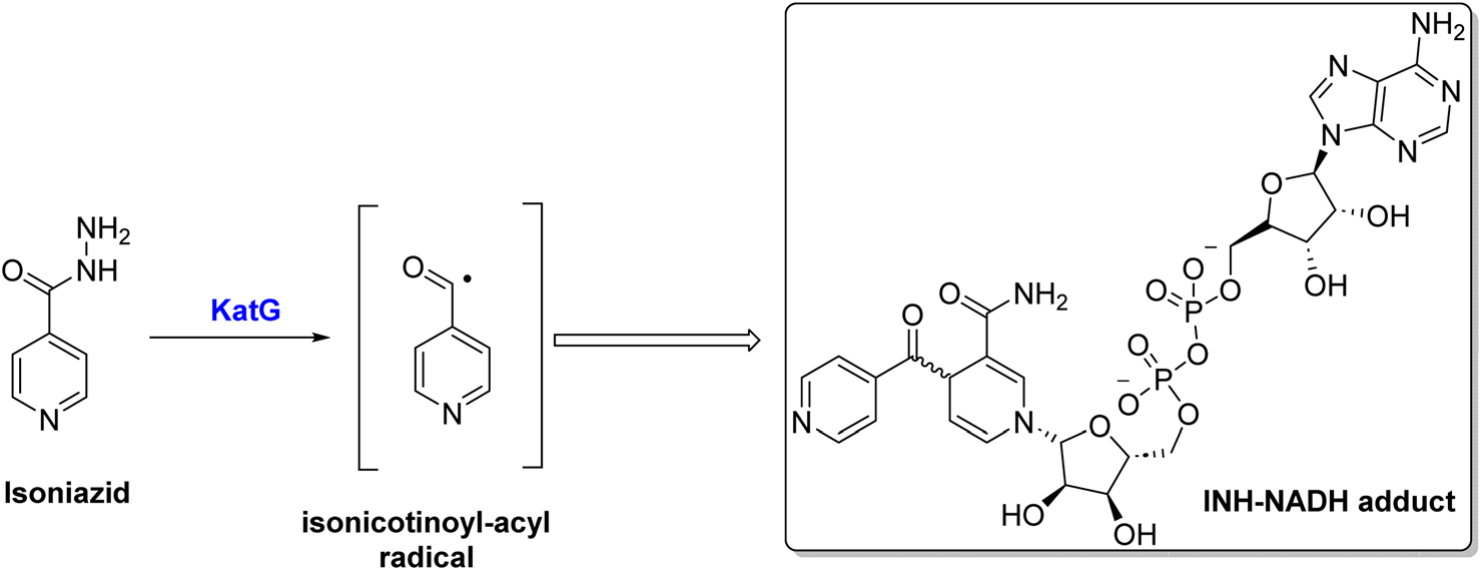
Activation of isoniazid *via* KatG enzyme.

Due to the unique cell envelope of tubercle bacilli, TB treatment is more challenging than other microbial infections. Cell envelopes consist of a defensive layer composed of mycolic acid, which is a saturated chain of β-hydroxy fatty acids with an α-alkyl side chain ^7^. An essential component of mycolic acid synthesis is the enoyl ACPR reductase (InhA), which reduces 2-trans enoyl-acyl carrier proteins (ACPs) in a NADH-dependent manner.^8^ Inhibiting the activity of the catalase-peroxidase enzyme KatG, isoniazid (INH) I inhibits InhA through its conversion into active form, INH-NADH, when it reacts with a NAD/NADH species^9^ (Scheme 1). The INH-NADH molecule acts as an inhibitor of the enoyl ACP reductase (InhA), which is responsible for mycolic acid production. This inhibition leads to the disruption of the cell wall and ultimately results in cell death, effectively inhibiting tuberculosis.^10–12^ InhA is the only essential gene encoded by *inhA*, but mutations in several other genes have been identified as mediating resistance to INH.^13^ Therefore, it is of utmost importance to note that mutations that abrogate KatG activity, which is prevalent in many clinical strains worldwide, prevent INH from being activated into active forms, preventing it from being utilized.^14^ It is, therefore, imperative that new compounds be developed that target InhA and can be used against multidrug-resistant strains of *M. tuberculosis*.^15^

Three pockets are present in the InhA active site, as revealed by the binding mode of its inhibitors. As the cofactor NAD is composed of Tyr158 residues and ribose moiety, it forms its first pocket. Two hydrophobic pockets accommodate several inhibitor moieties in the second. A hydrophilic side of the third pocket forms the home for the phosphate group of NAD, while a hydrophobic side consists of Ala198 and Ile202.^16^

**Fig. 1:**
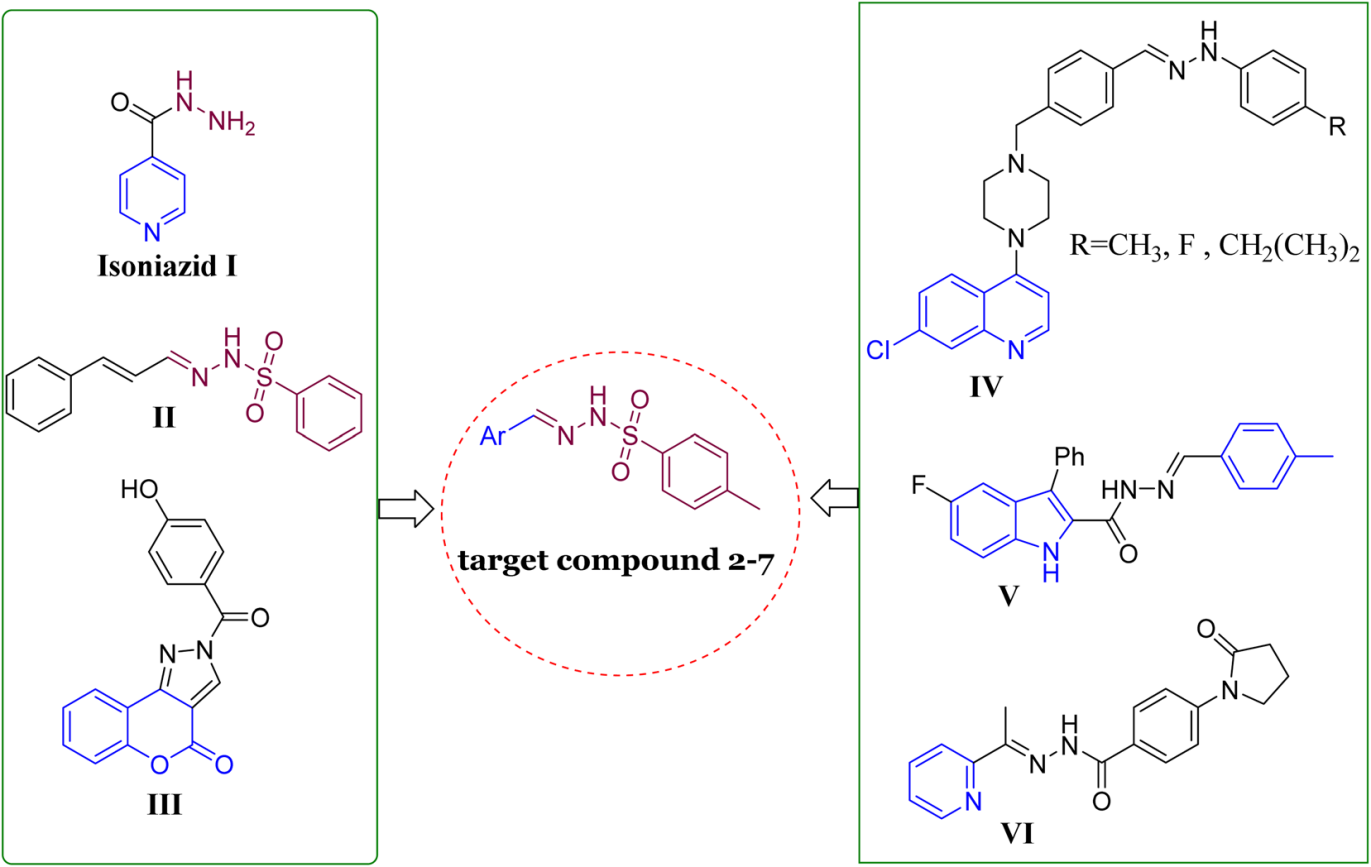
Structures of representative hybrids of bioactive cores I–VI and our newly designed compounds 2–7.

Hydrozones (HYDs) contain an azomethine group (HC=N) and have been extensively studied for their structural properties and possible applications in medicinal chemistry.^17^ Many biological activities have been shown to be enhanced by sulfonyl hydrazones, including antiviral,^18^ antimicrobial,^19^ acetylcholinesterase and α-glycosidase inhibitory activities,^20,21^ anticancer,^22^ and the ability to inhibit some other enzymes.^23,24^ Developed aryl sulfonyl HYDs have been the focus of a lot of research recently in order to find new and more effective antitubercular agents with fewer side effects, such as compound II (Fig. 1).^25^ InhA is considered to be the main molecular target of *N*-aryl sulfonyl hydrazone derivatives. Therefore, it is crucial to undertake additional investigations to thoroughly validate this hypothesis by examining their mechanism of action.^26–28^

On the other hand, the main moiety in heterocyclic molecules found in a variety of synthetic drugs, pharmaceuticals, bioactive natural products, and agrochemicals nowadays is either nitrogen or oxygen.^29^ The potency of drugs has been greatly increased by heterocyclic compounds that contain nitrogen and/or oxygen, such as coumarin, quinoline, pyridine, and/or pyridine. The pharmacological properties of these compounds span the gamut, including antibacterial, antileishmanial, anticonvulsant, anti-HIV, anticancer, and particularly anti-tuberculosis effects.^30–36^ Different types of these substances have been studied for their ability to fight against mycobacterial infections. A variety of coumarin compounds have become important in the search for new antimycobacterial activities,^37,38^ such as compound III.^39^ In 2020, the researchers highlighted the significance of quinoline hydrazone derivatives and their effectiveness against tuberculosis, *e.g.*, compound IV (Fig. 1).^40^ Moreover, Cihan-Üstünda and coworkers^41^ developed and tested new indole derivatives that showed promising results in inhibiting *Mycobacterium tuberculosis*, with compound V being the most potent with MIC = 25 μg mL^−1^. In another study, a novel series of pyridine derivatives were developed and explored for *in vitro* anti-tubercular action.^42^ Compound VI (Fig. 1) exhibited the most active against *Mycobacterium tuberculosis* with an MIC value of 14 ± 7 μM.

On the basis of these observations and as part of our continuing interest in the search for new compounds as antitubercular agents, we have designed, synthesized, and characterized a series of heterocyclic compounds-sulfonylhydrazone hybrids, whereas the 4-methylphenyl core is expected to occupy the hydrophobic pocket and form Pi–Pi stacking with Tyr158. The sulfonylhydrazide functional group may serve as a spacer that has been modified to facilitate hydrogen bonding interactions with essential amino acid residues.

**Fig. 2:**
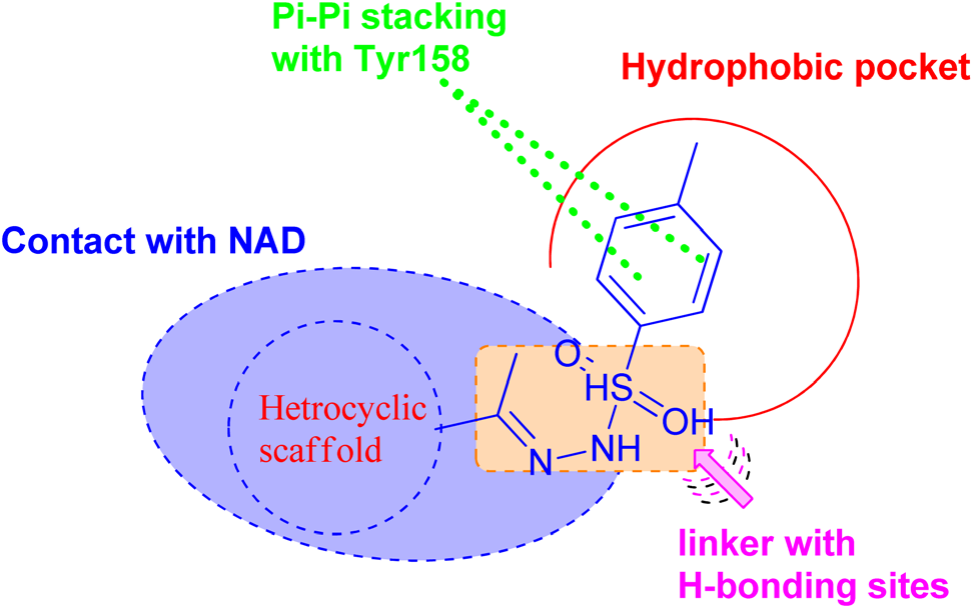
Pharmacophoric features for the most active molecules and binding interaction with the active site of InhA.

In addition, the heterocyclic scaffold can form a polar contact with the NAD cofactor (Fig. 2). The synthesized derivatives were tested for their activity toward *M. tuberculosis* and *M. abscessus*. The most potent counterparts were investigated for their inhibitory action on the *Mycobacterium tuberculosis* InhA enzyme. Molecular docking and molecular dynamic simulation studies were done to explore the plausible binding mode for the promising hits within the InhA active sit. Predictions have been made on the physicochemical and pharmacokinetic features of the promising candidates.

The work presents a series of benzenesulfonohydrazide-tethered heterocyclic compounds designed as potential novel anti-mycobacterial candidates targeting the InhA enzyme, which is crucial for the survival of *M. tuberculosis*. The study emphasizes the innovative approach of synthesizing both non-fused and fused heterocycles, including pyridine, coumarin, quinoline, and indole motifs, which exhibit significant bactericidal activity against *M. tuberculosis*. In addition, these molecules will be evaluated for the ability to penetrate human macrophages and inhibit mycobacterial biofilm formation, addressing a critical gap in current tuberculosis treatment strategies and thereby contributing to the ongoing efforts in anti-tuberculosis drug discovery.

## 2. Results and discussion

### 2.1. Chemistry

**Scheme 2:**
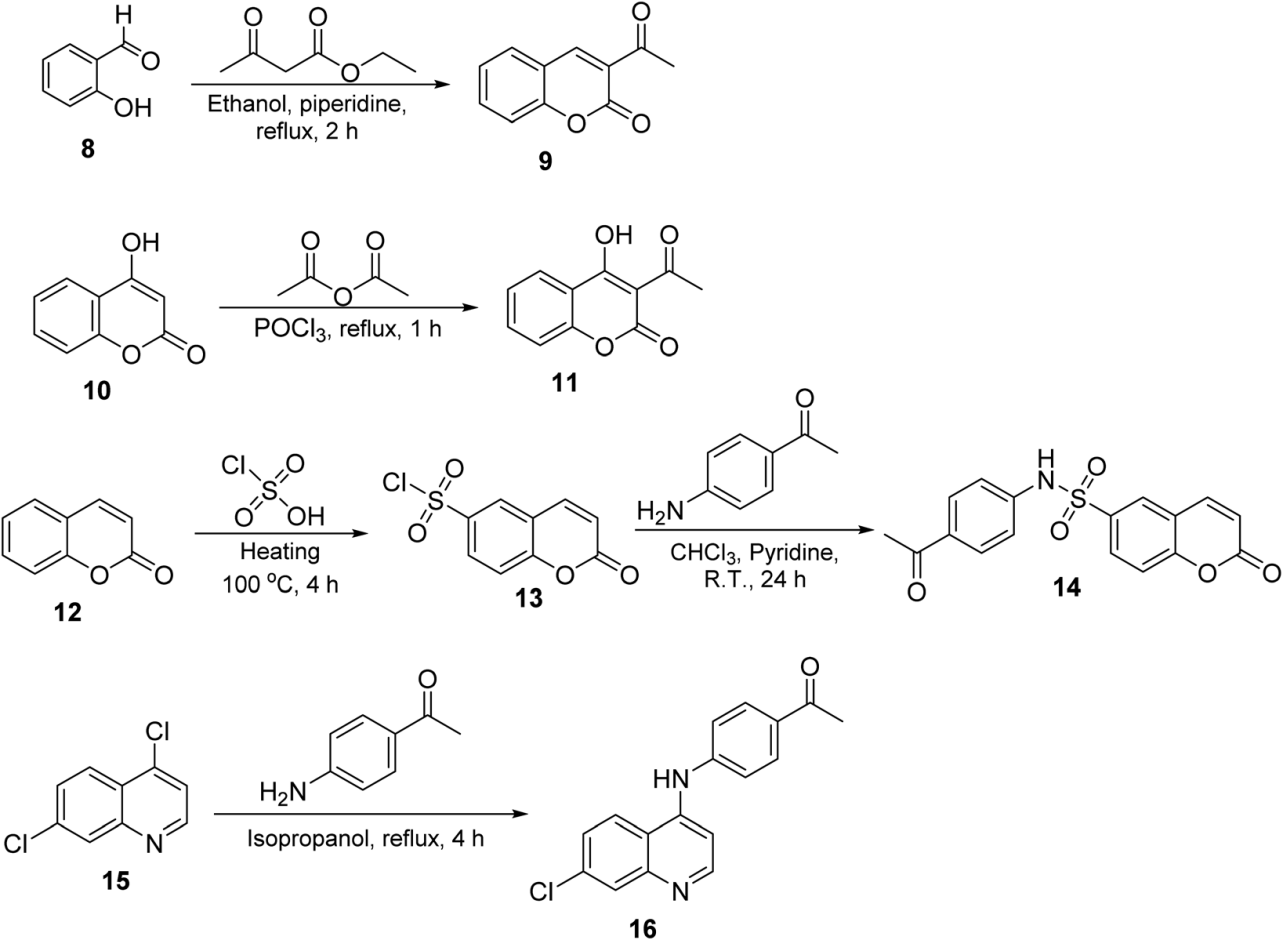
Synthesis of the target compounds 2–7.

**Scheme 3:**
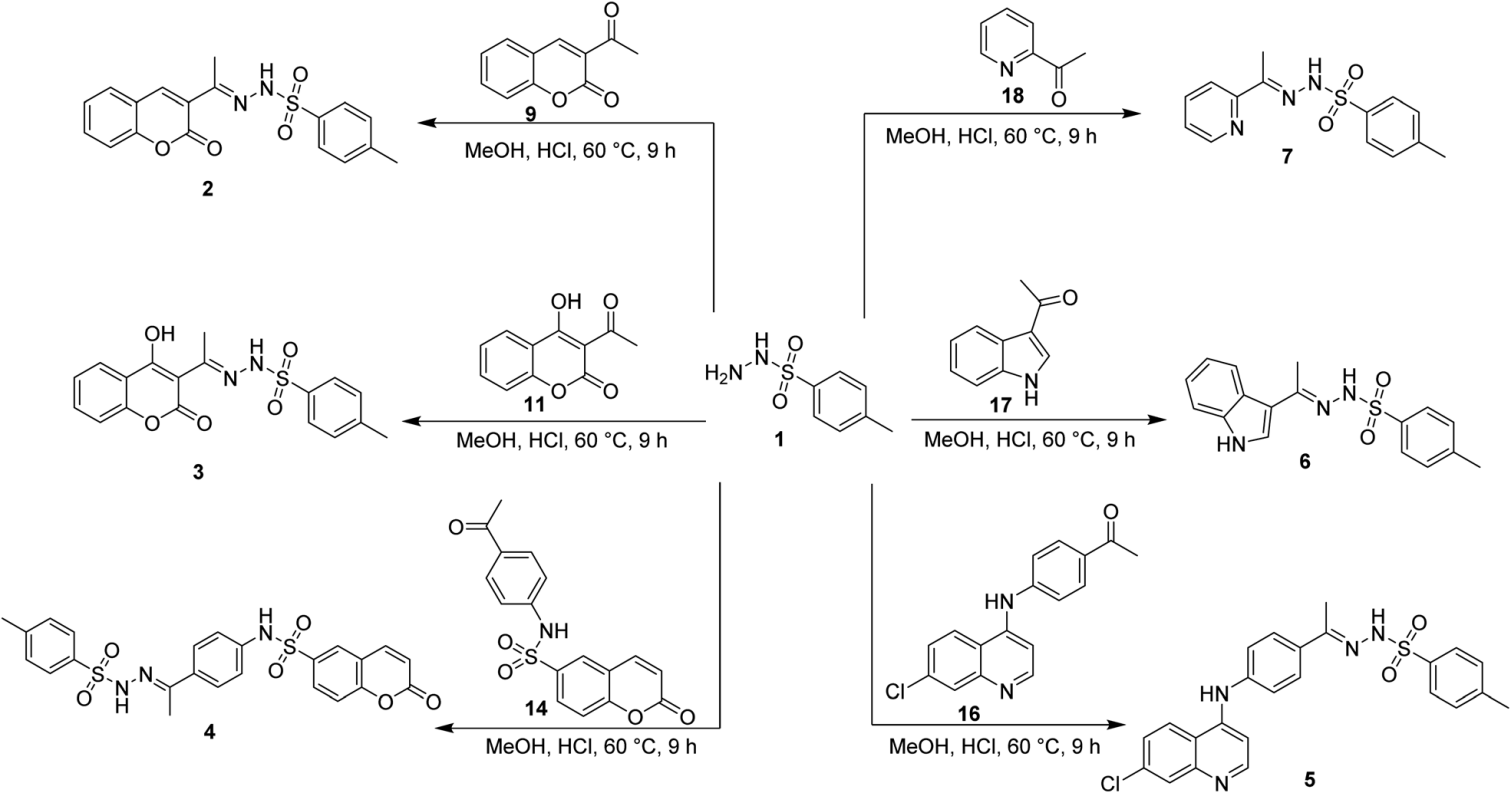
Synthesis of the target compounds 2–7.

The synthesis of target compounds 2–7 involves the preparation of key intermediates, specifically the acetyl heterocycles (9, 11, 14, and 16), as illustrated in Scheme 2. Following this, the target sulfonyl hydrazones 2–7 were synthesized through a condensation reaction between p-toluene sulfonylhydrazide and various heterocyclic compounds, including 3-acetylcoumarin (9), 4-hydroxy-3-acetylcoumarin (11), *N*-(4-acetylphenyl)-2-oxo-2*H*-chromene-6-sulfonamide (14), 1-(4-((7-chloroquinolin-4-yl)amino)phenyl)ethan-1-one (15), 3-acetylindole (17), and 2-acetylpyridine (18). This reaction was conducted in hot methanol with a small amount of hydrochloric acid, providing an efficient pathway to these sulfonyl hydrazones, which may possess significant biological activity (Scheme 3). To confirm their structures, the newly created target derivatives were analyzed using analytical and spectral techniques such as ^1^H NMR and ^13^C NMR.

### 2.2. Biological activity

#### 2.2.1. Identification of compounds with antituberculosis activity and determination of their cytotoxicity

Six target compounds of heterocycles derived from benzenesulfonyl hydrazide were assessed for their potential bactericidal effects against *M. tuberculosis* and fast-growing mycobacteria, including the highly drug-resistant opportunistic pathogen *M. abscessus*.^43^ All the molecules discussed in this study underwent initial screening at a uniform 125 μg mL^−1^ concentration. Three derivatives (3, 6, and 7) demonstrated growth inhibitory effects on *M. tuberculosis*, while none of them exhibited inhibitory effects on *M. abscessus*. Subsequently, the minimal inhibitory concentration (MIC) of the tested compounds that effectively inhibited the growth of *M. tuberculosis* was identified. As demonstrated using the microplate alamar blue assay (MABA), compounds 3, 6, and 7 inhibited the metabolic activity of mycobacteria at concentrations 30, 125, and 8 μg mL^−1^, respectively (Table 1). To ensure their safety profile, the two highly effective molecules (3 and 7) were evaluated for their cytotoxic effects on a mouse fibroblast L929 cell line in accordance with international guidelines (ISO 10993-5:2009(E)) through the MTT assay (Table 2).^44^ Considering the ratio of minimum inhibitory concentration (MIC) to half-maximal inhibitory concentration (IC_50_), compounds 3 and 7 were chosen for additional biological evaluations.

**Table 1:** MIC values [μg mL^−1^] of tested compounds (2–7) against *M. tuberculosis* and *M. abscessus*, as well as the bactericidal concentration^a^

Compound	MIC [μg mL^−1^]		Bactericidal concentration
	*M. tuberculosis*	*M. abscessus*	
2	>125	>125	ND
3	30	>125	100
4	>125	>125	ND
5	>125	>125	ND
6	125	>125	ND
7	8	>125	60

a ND: not determined.

**Table 2:** IC_50_ values [μg mL^−1^] of compounds 3 and 7 for their cytotoxic effect on a mouse fibroblast L929 cell line, as well as the calculated ratio of IC_50_/MIC

Compound	IC_50_ (L929 cells)	IC_50_/MIC (MTB)
3	625	21
7	78	10

#### 2.2.2. Evaluation of the bactericidal efficacy of selected compounds

**Fig. 3:**
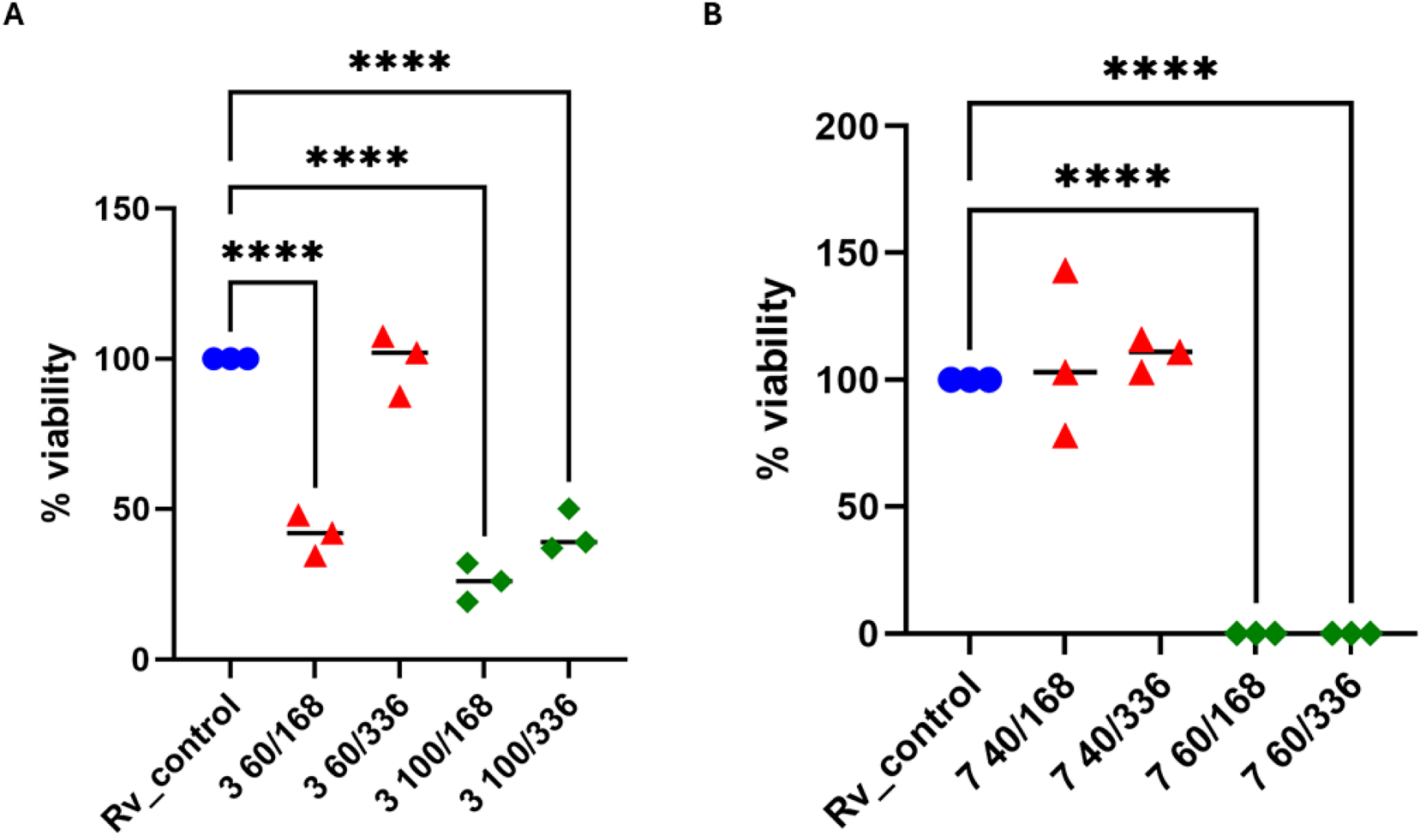
Bactericidal effect of sulfonyl hydrazone derivatives. (A) Compound 3 was used at concentrations of 60 and 100 μg mL^−1^ for 7 and 14 days. (B) Compound 7 was used at concentrations of 40 and 60 μg mL^−1^ for 7 and 14 days. The quantity of viable bacteria was evaluated through colony-forming unit (CFU) analysis. A standard one-way analysis of variance (ANOVA) test was utilized to compare the untreated *M. tuberculosis* control group (Rv_control) with bacilli treated with 3 and 7 compounds at specified time intervals. The corrected *p*-value was less than 0.0001 for paired comparisons denoted by asterisks. The statistical evaluation and visualization were conducted utilizing GraphPad Prism 9 version 9.3.1.

To assess the killing efficacy of the most active compounds against *M. tuberculosis*, their bactericidal effects were measured. The bactericidal potency of compounds 3 and 7 was evaluated by examining the survival rate of tubercle bacilli following exposure to these compounds in a broth culture for durations of 7 and 14 days. The quantity of living bacteria was assessed by counting the colony-forming units (CFU). Tuberculosis-causing bacteria were grown in a nutrient-rich environment with or without specific substances, 60 and 100 μg mL^−1^ (compound 3) or 40 and 60 μg mL^−1^ (compound 7), to determine the concentration at which the viability of *M. tuberculosis* was notably reduced, the mycobacteria were exposed to various compounds. Subsequently, the mycobacteria were plated on solid media (7H9/OADC) immediately following compound addition, as well as after 7 and 14 days of incubation. After a three-week incubation period, the quantity of viable bacilli (CFU) was enumerated and contrasted with an untreated control group (Fig. 3a and b). Compound 3 in concentration of 60 μg mL^−1^ Reduced the survival rate of *M. tuberculosis* by about 50% in 7 days (*p* < 0.0001); however, the number of viable bacteria increased again after 14 days. The concentration of 100 μg mL^−1^ of compound 3 Reduced the survival rate of tubercle bacilli by about 80 and 60% after 7- and 14 days incubation (*p* < 0.0001), respectively (Fig. 3a). Compound 7 in concentration 40 μg mL^−1^ did not decrease the viability of *M. tuberculosis* incubated up to 14 days. On the other hand, the same compound in concentration 60 μg mL^−1^ decreased the viability of tubercle bacilli by about 99%, both tested after 7 and 14 days of incubation (*p* < 0.0001) (Fig. 3b).

#### 2.2.3. Evaluation of the antimycobacterial efficacy of specific compounds within human macrophages

Tuberculosis (TB) is an air-born disease, and the infection is initiated when the pathogen reaches the alveolar compartments of the host. As with any other pathogens, after infection, *M. tuberculosis* is phagocytes by alveolar macrophages, professional phagocytes responsible for the intracellular killing of bacteria. However, tubercle bacilli can survive and multiply inside human macrophages by affecting their response to infection. Therefore, the antituberculosis drugs should be able to penetrate human macrophages without causing their lysis. To examine the activity of the tested molecules against intracellular bacteria, we assessed their toxicity to human macrophages at 2× and 4× MIC concentrations. Human macrophages (MDMs) were derived from monocytes obtained from buffy coats of healthy human blood donors through a process of differentiation.

**Fig. 4:**
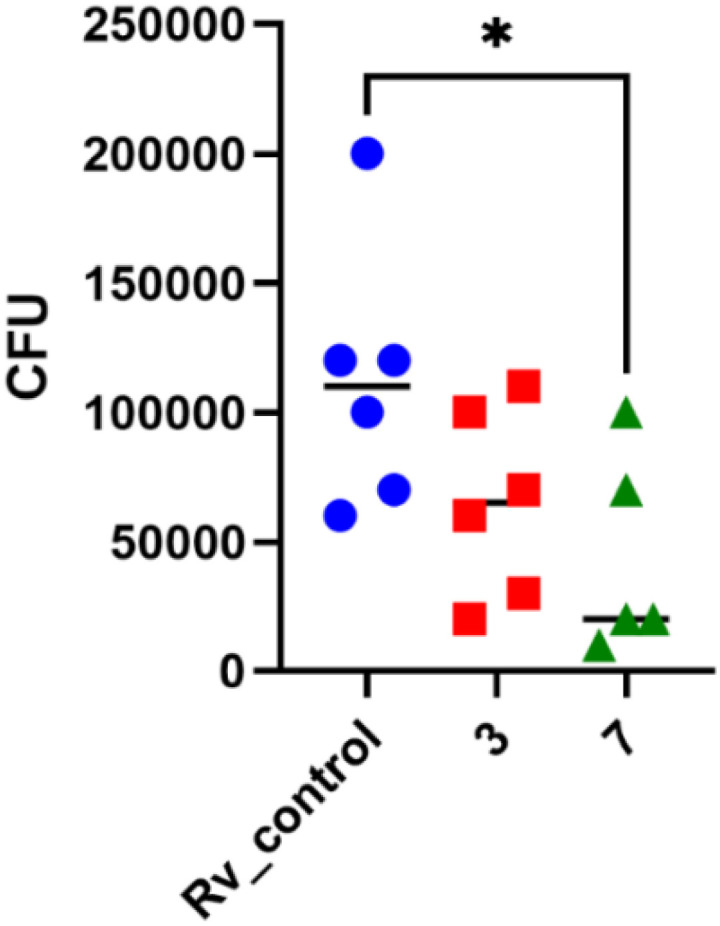
Human monocyte-derived macrophages (MDMs) were infected with *M. tuberculosis* and exposed to compounds 3 and 7 at a concentration of 2 times the minimum inhibitory concentration (MIC). A standard one-way analysis of variance (ANOVA) was conducted to compare the quantity of intracellular *M. tuberculosis* in the control group with that in the bacilli treated with compounds 3 and 7. The adjusted *p*-value indicated statistical significance (*p* = 0.0358) only for compound 7. The statistical analysis and graphical representation were performed using GraphPad Prism 9 version 9.3.1.

In 2× MIC concentration, both compounds presented low cytotoxicity against MDMs. However, 4× MIC concentration of compound 3, but not 7, appeared to be toxic to the host cells. Afterward, both substances were evaluated for their effectiveness against tuberculosis bacteria present in human macrophages. To evaluate the effectiveness of the studied compound on *M. tuberculosis* inside human macrophages, tubercle bacilli were used to infect the MDMs at a ratio of 1 : 10. Following 2 hours of phagocytosis, the bacteria outside the cells were removed, and the remaining bacilli attached to the cell membrane were eliminated using gentamicin. The macrophages containing *M. tuberculosis* were then treated with the compounds at a concentration of twice the minimum inhibitory concentration (MIC) and left to incubate for 48 hours. The quantity of viable bacilli located inside cells was assessed by counting colony-forming units (CFU). Both compounds caused a decrease in the number of live bacteria in MDMs in comparison to the control group that did not receive any treatment. The statistical significance of the effect was observed solely in the instance of compound 7, with a *p*-value of 0.0358, suggesting its ability to affect the intracellular viability of tubercle bacilli (Fig. 4).

#### 2.2.4. Evaluating the efficacy of substances on mycobacterial biofilms

**Fig. 5:**
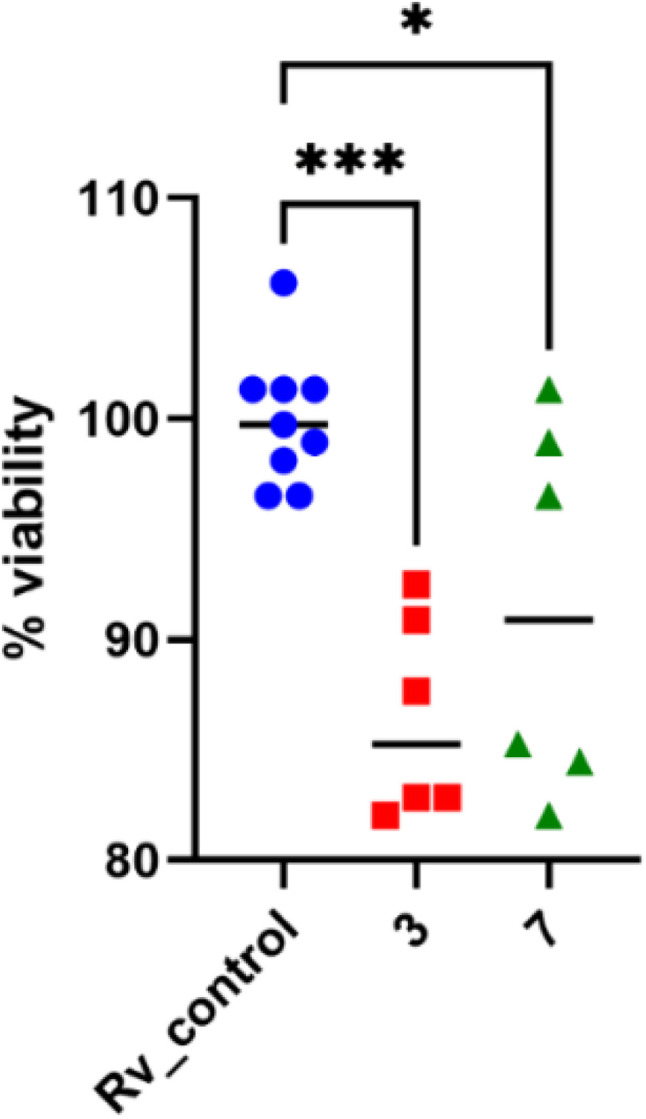
The impact of sulfonyl hydrazon derivatives on established biofilms consisting of *M. tuberculosis* was assessed. Statistical comparisons were conducted through standard one-way ANOVA analysis. Blue dots in the figures represent the absence of compound control. Detailed *p* values are provided in the accompanying text. The statistical analysis and graphical representation were performed using GraphPad Prism 9 version 9.3.1.

Mycobacteria, similar to numerous other bacterial strains, possess the capacity to attach to surfaces and form biofilms.^45,46^ Tubercle bacilli have the ability to form a biofilm in a laboratory setting, but Ojha *et al.* have not determined its significance in the development of tuberculosis.^47^ Drugs are often ineffective against the bacteria in biofilms due to limited penetration throughout the biofilm structure. The novel drug candidates must demonstrate bactericidal activity against planktonic bacilli residing either intracellularly or extracellularly, as well as against bacteria capable of forming biofilms. To evaluate the compounds' ability to inhibit or disrupt mycobacterial biofilms, which are often associated with drug tolerance, biofilm formation assays were conducted. The compounds 3 and 7 were assessed for their effectiveness against the biofilm produced by *M. tuberculosis* in Sauthon's medium, following the methodology outlined in the Experimental section. The biofilm's growth medium was exchanged with a new medium containing verified compounds at concentrations of 2 times the minimum inhibitory concentration (MIC) and 4 times the MIC. The biofilm was then incubated for an additional 48 hours. The assessment of tubercle bacilli viability in biofilm cultures, using resazurin, enabled the differentiation of the bactericidal impact of the tested substances on *M. tuberculosis* biofilm compared to control cultures. The viability of *M. tuberculosis* biofilms was reduced by around 15% and 10% when sulfonylhydrazone derivatives (3 and 7) were present at a concentration of 4 times the minimum inhibitory concentration (MIC). This decrease in viability was statistically significant, with *p*-values of 0.0003 and 0.0148, respectively (Fig. 5).

#### 2.2.5. Inhibition of *Mycobacterium tuberculosis* (MTB) InhA enzyme

To elucidate the potential mechanism of action, we focused on the two compounds that exhibited the most potent activity against MTB: the coumarin-based derivative (3) and the pyridine-based derivative (7). Our investigation aimed to determine their specific effects on mycobacterial cells. As part of this exploration, we evaluated the compounds' efficacy in inhibiting the InhA enzyme, a critical target in mycobacterial metabolism (Table 3). This approach allowed us to gain deeper insights into how these promising molecules might exert their antimycobacterial effects at the molecular level. INH was selected to serve as a comparative positive control.

**Table 3:** InhA inhibition effect of the compounds 3 and 7

Compound	IC_50_ (μM)
3	1.62 ± 0.69
7	0.91 ± 0.03
Isoniazid	0.24 ± 0.01

According to Table 3, compound 7 effectively inhibited the InhA enzyme at micromolar concentration, with an IC_50_ value of 0.91 ± 0.03 μM. Compound 3 had reduced activity against InhA, with an IC_50_ value of 1.62 ± 0.69 μM.

The differential antimicrobial activity of derivative 7 towards *M. tuberculosis* and *M. abscessus* could be attributed to certain factors. While both species belong to the Mycobacterium genus, their InhA enzymes share only 75–80% sequence identity, which should lead to structural differences affecting the binding of the potential inhibitors. These variations could alter the binding site's hydrophobicity, electrostatic properties, or shape, potentially resulting in different binding affinities for compound 7. Furthermore, *M. abscessus* is known to have a more complex and less permeable cell wall than *M. tuberculosis*, which may restrict the molecule's ability to reach its intracellular target. Additional factors, such as specific efflux pump systems and metabolic differences, could also potentially be responsible for the observed variance in antimicrobial activities.

### 2.3. Molecular docking

**Fig. 6:**
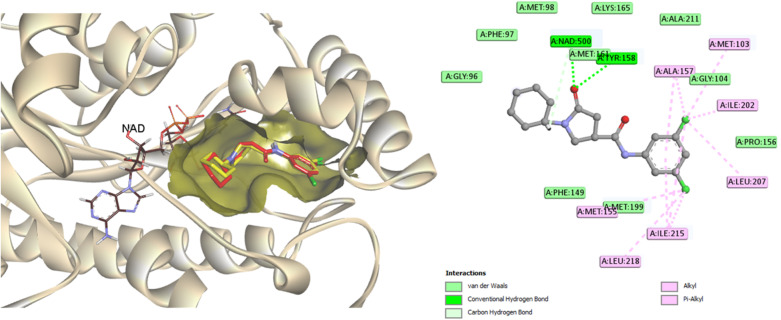
A two-dimensional interaction map and three-dimensional overlay plot were generated for the InhA protein active site (PDB ID: 4TZK)^47^ comparing the co-crystallized ligand (in red) with the re-docked ligand (in yellow), revealing a root mean square deviation (RMSD) value of 0.5699 Å.

Molecular docking analysis was performed to enhance comprehension of the potential binding mechanisms of the potent compounds 3, 6, and 7 with InhA. The docking outcomes of the studied compounds were evaluated against the co-crystallized ligand found in the PDB ID: 4TZK,^48^ the reference drug utilized in this experiment has been previously demonstrated to exhibit strong inhibitory activity against InhA. During the docking procedure, the NAD cofactor was maintained in proximity to the active site. The initial stage of validating the docking process involved re-docking the co-crystallized ligand. The redocked ligand exhibits an RMSD value of 0.5699 Å, indicating a precise prediction of its correct positioning, as illustrated in Fig. 6. Analysis of the ligand interaction within the co-crystallized structure revealed the presence of multiple hydrophobic contacts and two hydrogen bonds, specifically at residues Tyr158 and Met161.

**Fig. 7:**
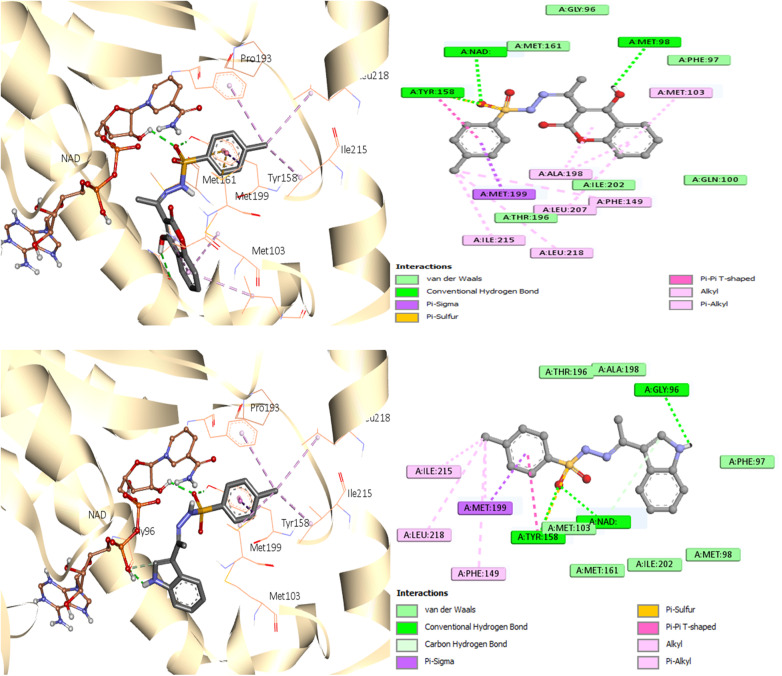
Molecular interactions between InhA residues and ligands 3 and 6 on a 2D and 3D scale.

As seen in Fig. 7, during the tests, the active site of the NAD molecule was occupied by the compounds tested. It was observed that Tyr158 and the sulfonyl hydrazine linker of these compounds form hydrogen bond through the oxygen atom of the sulfonyl group. The phenyl ring of compounds 3 and 6 stacked in π interactions with Tyr158 and Met199. It was also discovered that there is a hydrophobic interaction between the aromatic moiety of 3 (4-hydroxycoumarin) and 6 (indole) and the active site of InhA *via* a hydrophobic pocket (Met103, Ala198, and Leu207). Moreover, an H-bond was formed between Met 98 and the hydroxy on coumarin for compound 3 and between Gly96 and the indole NH for compound 6.

**Fig. 8:**
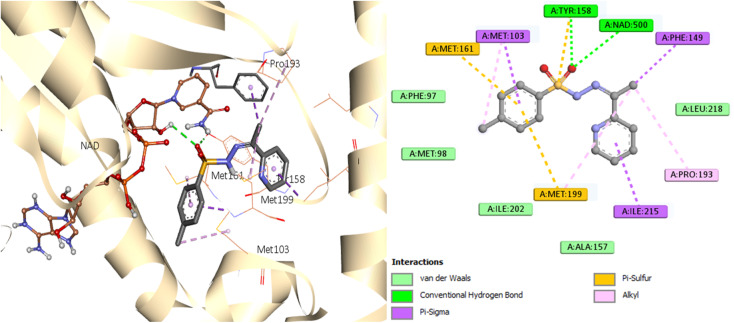
Molecular interactions between InhA residues and ligand 7 on a 2D and 3D scale.

A similar reaction took place with compound 7, which occupied the NAD molecule in contact with the active site. The sulfonyl hydrazine linker forms a hydrogen bond through its oxygen with Tyr158, alongside sulfur–π interaction with its sulfur atom (Fig. 8).

The phenyl ring showed π interactions with Met103, Met161, and Met199. It was also discovered that there is a hydrophobic interaction between the second aromatic moiety and the InhA's active site (Phe149, Pro193, Met199, and Ile215) of the hydrophobic pocket.

The most firmly bound derivatives among the tested compounds showed binding affinity of −8.3, −8.7, and −9.2 kcal mol^−1^, which is approximately similar to the binding affinity of the co-crystallized ligand (−9.5 kcal mol^−1^). By illuminating the patterns of interaction of newly synthesized compounds, these findings may provide a strong basis for the development of antituberculosis drugs.

### 2.4. Molecular dynamic simulations

MD study on the ideal pose for molecule 7 inside the active region of the InhA protein was carried out to examine the stability of the complex structure. Consequently, an assessment and comparison were conducted between the 7 complex system and the apoprotein using a 100 ns MD simulation conducted at ambient temperature. First, consistent temperatures, constant potential energy, and constant pressure were observed throughout the simulation, confirming the convergence of the complex system (Fig. S1†).

**Fig. 9:**
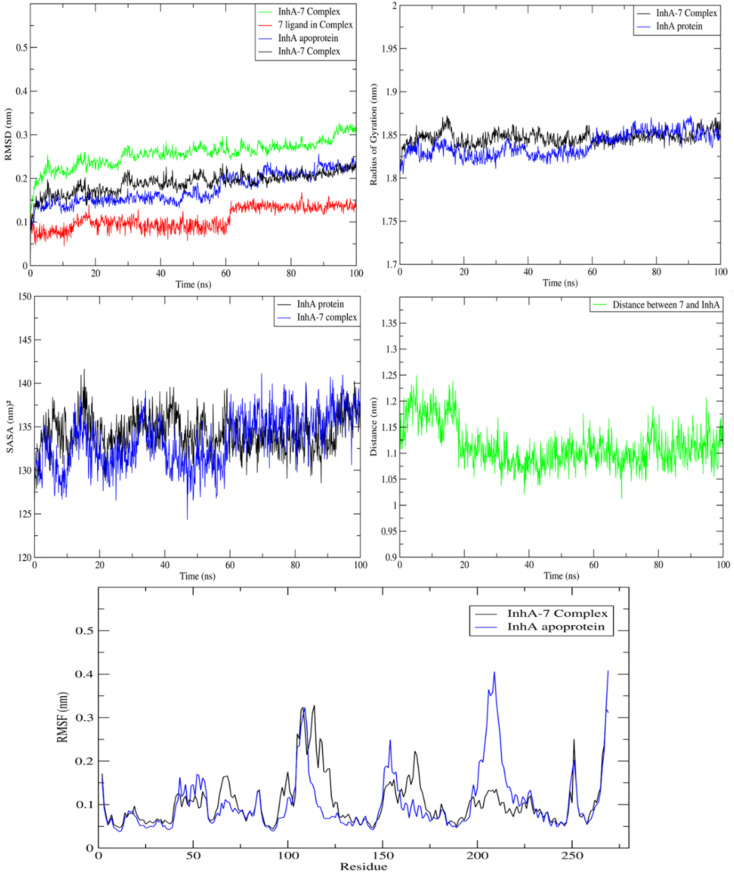
Plots of ligand-receptor distance, SASA, radius of gyration RMSD and RMSF for InhA protein with and without 7.

Observing the drift of RMSD along the trajectories, the difference between the InhA protein in the presence and absence of compound 7 was examined. Because of the InhA protein's stabilization, Fig. 9 demonstrated a similar pattern between the complex RMSD values and the apoprotein one. Additionally, it was clear that the complex's SASA and radius of gyration were similar to those of the apoprotein, indicating that there was no induction of conformational changes and formation of a stable complex with 7. Furthermore, during the MD, the average distance of 7/InhA protein did not change. Additionally, the reduced draft found in the complex's RMSF relative to the free InhA protein further confirmed the stability of the 7-InhA protein complex (Fig. 9).

**Fig. 10:**
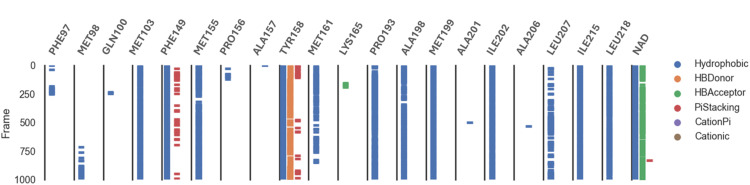
Various interacting amino acids with the sorts of interaction with 7 into InhA protein active cavity.

Analysis of the MD trajectories for compound 7 simulations with the InhA receptor revealed stable binding interactions, including two robust hydrogen bonds and consistent hydrophobic interactions (Fig. 10). Specifically, two distinct hydrogen bonds were observed: one with Tyr158 and another with NAD. Additionally, intermittent π-stacking interactions were noted with Phe149 and Tyr158. Table 4 summarizes the various binding energies of the complex, calculated using the MMPBSA method. These results demonstrate how the observed interactions contribute to the overall stability of the compound–receptor complex.

**Table 4:** Free binding energies of 7 with InhA protein in kJ mol^−1^

Binding energies	kJ mol^−1^
Electrostatic energy	−23.935 ± 2.415
Polar solvation energy	54.240 ± 15.552
SASA energy	−15.247 ± 0.722
van der Waal energy	−121.296 ± 10.761
Δ*G*	−106.239 ± 15.899

## 3. Conclusions

In summary, this study prepared a series of small molecules based on non-fused and fused heterocycles (pyridine, coumarin, quinoline, and indole) tethered with benzenesulfonohydrazide as potential antitubercular candidates. The developed molecules were assessed for their potential bactericidal effects against *M. tuberculosis* and *M. abscessus*. Coumarin-based derivative (3) and pyridine-based derivative (7) showed MIC values of 30 and 8 μM, respectively, against *M. tuberculosis*. Further tests on macrophage and antibiofilm activity were performed for the two most active compounds. Mycobacteria could not grow inside human macrophages, but there was a limited reduction in the viability of biofilm-forming mycobacteria with both compounds. A strain overexpressing InhA was also shown to be sensitive to these two compounds with IC_50_ values of 1.62 ± 0.69 and 0.91 ± 0.03 respectively. To rationally understand how the compounds interact with the enzyme, docking studies revealed hydrogen bond interactions with Tyr 158 to be critical for binding InhA, as well as hydrogen bonds with Met161. Based on molecular docking simulations, this compound was found to have similar binding affinities and interactions to previously reported InhA inhibitors, emphasizing the importance of hydrogen and halogen bonds, as well as other interactions that contribute to stability within the InhA active site. The MD analysis also confirmed the binding mode observed in docking studies, which supports the stability of InhA-7. The results of this study pave the way for several promising research directions and optimization opportunities. Future investigations could focus on further modifying the most potent molecules (3 and 7) to enhance their efficacy and selectivity against mycobacterial InhA. Additionally, exploring combination therapy approaches could lead to the development of more effective treatment regimens by evaluating potential synergistic effects between these compounds and existing anti-tuberculosis drugs. Moreover, conducting *in vivo* animal studies is a crucial next step to assess the safety profiles, pharmacokinetic properties, and therapeutic efficacy of the most promising molecules in relevant tuberculosis models. These avenues of research have the potential to advance significantly the treatment of mycobacterial infections.

## 4. Experimental

### 4.1. Chemistry

The melting points of all samples were determined using the Electrothermal IA 9000 instrument without any corrections. The nuclear magnetic resonance spectra, specifically the ^1^HNMR (400 MHz) and ^13^CNMR spectra (100 MHz), were obtained using a Bruker spectrometer. TMS was used as the internal standard. The reactions were monitored using thin-layer chromatography (TLC) with silica gel and aluminum sheets 60 F_254_ from Merck. The eluent used was a mixture of chloroform and methanol (9.5 : 0.5 v/v), and the TLC plates were sprayed with an iodine-potassium iodide reagent. Compounds 3, 4, 6, and 7 were previously synthesized.^49–53^

#### 4.1.1. General procedure for the preparation of target compounds (2–7)

Three catalytic drops of hydrochloric acid were added to a mixture of *p*-toluenesulfonyl hydrazide 1 (1 mmol) and the appropriate acetyl derivatives (1 mmol) in methanol (15 mL). The reaction mixture was then heated at 60 °C for 9 hours. The resulting solid was separated by filtration and rinsed with chilled methanol, followed by purification through recrystallization using isopropanol, resulting in the formation of compounds 2–7.

#### 4.1.2. 4-Methyl-*N*′-(1-(2-oxo-2*H*-chromen-3-yl)ethylidene)benzenesulfonohydrazide (2)

Yellow crystals; mp 188–189 °C; yield (83%);^1^H NMR (400 MHz, DMSO-d_6_) *δ*: 2.13 (s, 3H, CH_3_), 2.37 (s, 3H, CH_3_), 7.32-7.42 (m, 4H, H–Ar), 7.58-7.64 (m, 1H, H–Ar), 7.76-7.82 (m, 3H, H–Ar), 7.86 (s, 1H, H4 of coumarin), 10.69 (brs, 1H, NH); ^13^C NMR (100 MHz, DMSO) *δ* 16.41 (CH_3_), 21.08 (CH_3_), 115.99, 118.55, 124.77, 126.23, 127.54, 129.29, 129.60, 132.53, 136.13, 141.17 (Ar), 143.50 (C–SO_2_), 151.60 (C9 of coumarin), 153.37 (C=N), 159.00 (C=O); analysis for C_18_H_16_N_2_O_4_S, M. wt. (356.40), calcd: % C, 60.66; H, 4.53; N, 7.86; found: % C, 60.81; H, 4.49; N, 7.98.

#### 4.1.3. *N*′-(1-(4-((7-Chloroquinolin-4-yl)amino)phenyl)ethylidene)-4-methylbenzenesulfonohydrazide (5)

White crystals; mp 168–170 °C; yield (76%); ^1^H NMR (400 MHz, DMSO-d_6_) *δ*: 2.23 (s, 3H, CH_3_), 2.38 (s, 3H, CH_3_), 6.86 (d, 1H, *J* = 6.90 Hz, H of quinoline), 7.40 (d, 2H, *J* = 8.1 Hz, H–Ar), 7.48 (d, 2H, *J* = 8.4 Hz, H–Ar), 7.78-7.85 (m, 5H, H–Ar), 8.17-8.20 (m, 1H, H–Ar), 8.50 (d, 1H, *J* = 6.9 Hz, H2 of quinoline), 8.84-8.95 (m, 1H, H5 of quinoline), 10.65 (brs, 1H, NH), 11.31 (brs, 1H, NH); ^13^C NMR (100 MHz, DMSO) *δ*: 14.28 (CH_3_), 21.01 (CH_3_), 100.59 (C3 of quinoline), 116.11, 119.14, 124.89, 126.32, 127.36, 127.55, 128.83, 129.01, 129.48, 136.11, 136.21, 137.96, 138.37, 139.04 (Ar), 143.33 (C–SO_2_), 152.18 (C=N), 154.48 (CH_3_–C=N); analysis for C_24_H_21_ClN_4_O_2_S, M. wt. (464.97), calcd: % C, 62.00; H, 4.55; N, 12.05; found: % C, 62.19; H, 4.51; N, 12.13.

### 4.2. Biology

#### 4.2.1. MIC determination

*Mycobacterium tuberculosis* H37Rv and *Mycobacterium abscessus* strains were cultured in a nutrient-rich 7H9/OADC medium (Middlebrook, Difco, MD, USA). The medium was supplemented with different concentrations of the small molecules being tested in order to determine the minimal inhibitory concentration (MIC) values. The synthesized molecules were dissolved in DMSO and directly introduced to the growth medium at a concentration that never exceed 0.1% (vol/vol). This concentration did not have any impact on the growth of the bacilli. The Microplate Alamar Blue Assay (MABA test) developed by Franzblau *et al.* (1998)^54^ was used to determine the MIC value. The susceptibility of the examined strains was determined through a visual evaluation of the color, ranging from blue to pink. Controls consisting of wells containing simply bacteria, medium, or compounds were utilized in this experiment, and the MABA test was independently replicated three times.

#### 4.2.2. *In vitro* cytotoxicity assay

The international standard (ISO 10993-5: 2009(E)) applying L-929 cells and the MTT protocol was used to determine the cytotoxicity of tested chemicals. In addition, the cytotoxicity of the chosen molecules reported here was assessed against human monocyte-derived macrophages. However, in this instance, the cells were exposed to the tested compounds for a duration of 48 hours.

#### 4.2.3. Bactericidal effect

The viability of bacilli growing in the presence of tested molecules has been determined *via* calculating the optical density (OD_600_) for *M. tuberculosis* H_37_Rv culture and enumeration of the colony-forming units (CFU). The culture of *M. tuberculosis* H_37_Rv growing at 37°C in rich medium (7H9/OADC/0.05% Tween 80) was supplemented with tested chemicals in various concentrations (60 and 100 μg mL^−1^ for 3, 40 and 60 μg mL^−1^ for 7) at OD_600_ = 0.1. Measurement of optical density and enumeration of colony-forming units was conducted after 7 and 14 days. The culture of mycobacteria, supplemented or not with tested chemicals, was diluted in fresh medium and plated on 7H10/OADC on the first day of the experiment, after 7 and 14 days. The number of colonies (CFU) was quantified after an incubation period of three to five weeks at a temperature of 37°C.

#### 4.2.4. Biofilm formation

The biofilm of *M. tuberculosis* was created using the previously reported method with some modifications.^55^ The detailed protocol utilized was included in the ESI materials.†

#### 4.2.5. Preparation of human MDMs and evaluation of the bactericidal effect of the compounds on intracellularly growing tubercle bacilli

The commercially available, freshly prepared buffy coats from healthy human blood donors were exploited to isolate human monocytes using the published protocol.^56,57^ The MDMs were infected with tubercle bacilli, as previously described by Korycka-Machała *et al.*^58^ The number of CFUs was also determined as previously reported.^59^ All the procedures employed were included in the ESI materials.†

#### 4.2.6. Evaluation of InhA inhibition

InhA activity was assessed by a colorimetric assay that measured the oxidation of NADH at 340 nm in the presence of 2-trans-octanoyl-CoA, as reported^60^ (ESI materials†).

### 4.3. Molecular modeling and molecular dynamic simulation

The *M. tuberculosis* InhA protein's structural coordinates were obtained in pdb format from the RCSB PDB.^47^ Using AutoDock Tools,^61^ Marvin Sketch, AutoDock Vina,^62^ and Discovery Studio Visualizer,^63^ the docking study was carried out. In addition, CHARMM-GUI solution builder,^64–66^ GROMACS 2020.2 software,^67^ VMD molecular graphics program,^68^ and GROMACS g_mmpbsa tool^69^ were exploited in molecular dynamic simulation studies. The ESI materials† provide details on all the experimental steps and protocols.

## Data availability

The datasets supporting this article have been uploaded as part of the ESI.†

## Author contributions

T. A.-W.: investigation, data curation, formal analysis, funding acquisition; A. S.: conceptualization, formal analysis, methodology, resources, writing—original draft preparation; M. K.-M.: methodology, data curation; A. F. K.: methodology, investigation, formal analysis; M. A. S.: resources, formal analysis, writing—original draft preparation; H. A. A. I.: methodology, investigation, formal analysis; M. Ka.: methodology, data curation; B. D.: methodology, data curation; M. Ku.: methodology, data curation; W. M. E.: conceptualization, supervision, project administration, writing—review and editing; J. D.: conceptualization, formal analysis, supervision, writing—review and editing. All authors have read and agreed to the published version of the manuscript.

## Conflicts of interest

No potential conflicts of interest was reported by the author(s).

## Supplementary Material

RA-014-D4RA05616G-s001
