# Supplementary material for: Benzenesulfonohydrazide-tethered non-fused and fused heterocycles as potential anti-mycobacterial agents targeting enoyl acyl carrier protein reductase (InhA) with antibiofilm activity
Source: RSC Adv. 2024 Sep 23;14(41):30165–79. doi: 10.1039/d4ra05616g (PMC11418391; doi:10.1039/d4ra05616g)
Supplement: RA-014-D4RA05616G-s001 [file RA-014-D4RA05616G-s001.pdf]

Supplementary Information (SI) for RSC Advances.
This journal is © The Royal Society of Chemistry 2024

Benzenesulfonohydrazide-tethered non-fused and fused heterocycles as potential anti-mycobacterial agents targeting enoyl acyl carrier protein reductase (InhA) with antibiofilm activity

Tarfah Al-Warhi[1], Ahmed Sabt[2], Małgorzata Korycka-Machala[3], Asmaa F. Kassem [4], Moataz A. Shaldam[5], Hoda Atef Abdelsattar Ibrahim[6], Malwina Kawka[7], Bożena Dziadek[7], Magdalena Kuzioła[3,8], Wagdy M. Eldehna[5,9,*], Jarosław Dziadek[3 *]

[1] *Department of Chemistry, College of Science, Princess Nourah bint Abdulrahman University, Riyadh, Saudi Arabia*

[2] *Chemistry of Natural Compounds Department, Pharmaceutical and Drug Industries Research Institute, National Research Centre, Dokki, Cairo, 12622, Egypt.*

[3] *Laboratory of Genetics and Physiology of Mycobacterium, Institute of Medical Biology of the Polish Academy of Sciences, Lodz, Poland*

[4] *Department of Chemistry, College of Science and Humanities in Al-Kharj, Prince Sattam Bin Abdulaziz University, Al-Kharj, 11942, Saudi Arabia*

[5] *Department of Pharmaceutical Chemistry, Faculty of Pharmacy, Kafrelsheikh University, Kafrelsheikh 33516, Egypt*

[6] *Pediatric Department, Faculty of Medicine, Cairo University, Cairo, Egypt*

[7] *Department of Molecular Microbiology, Faculty of Biology and Environmental Protection, University of Lodz, Lodz, Poland*

[8] *Bio-Med-Chem Doctoral School of the University of Lodz and Lodz Institutes of the Polish Academy of Sciences, Lodz, Poland*

[9] *Department of Pharmaceutical Chemistry, Faculty of Pharmacy, Pharos University in Alexandria; Canal El Mahmoudia St., Alexandria 21648, Egypt*

**Corresponding author:* wagdy2000@gmail.com

jdziadek@cbm.pan.pl

1. Biological Evaluations

1.1. Biofilm formation

The biofilm of *M. tuberculosis* was developed as described previously with minor modifications [55]. *M. tuberculosis* was cultured to an OD_{600} = 1 in rich medium (7H9/OADC) supplemented with 0.05% Tyloxapol. The culture was then diluted 100 times in Sauton's medium and applied to the wells of a 24-well plate (2.5 ml/well), covered with a lid, protected with parafilm, and incubated in humidity at 37°C for 5 weeks. After obtaining the biofilm, the medium was replaced with fresh one with the addition of 0.1% casitone and the tested compounds at a concentration of 2x and 4x MIC, and it was further incubated at 37°C for 48 h. The viability of the bacilli was determined by fluorescence measurements (excitation: 550 nm, emission: 590 nm) in the presence of resazurin (375 µl of 0.02% resazurin per well) after 90 min of incubation using the multimode microplate reader SpectraMax® i3 (Syngen). The no compound control was used to represent 100% viability. The results were expressed as the percent viability compared to untreated *M. tuberculosis* biofilm.

1.2. Preparation of human MDMs, and evaluation of the bactericidal effect of the compounds on intracellularly growing tubercle bacilli

The commercially available, freshly prepared buffy coats (Regional Blood Donation Station, Lodz, Poland) from healthy human blood donors were used to isolate human monocytes using the published protocol [56, 57]. The nonadherent cells were removed from the cultures of differentiated human monocyte-derived macrophages (MDMs) by extensive washing, then the cells were rested overnight and incubated with culture medium supplemented with the various concentrations of the tested compounds. The macrophages' viability was assessed after 48 h of incubation using 3-(4,5-dimethylthiazol-2-yl)-2,5-diphenyltetrazolium bromide (MTT) (Sigma, St. Louis, MO, United States). Additionally, the MDMs were infected with tubercle bacilli at an MOI of 1:10 as described by Korycka-Machala *et al.* (2017) [58]. The extracellularly located bacteria were removed by washing with culture medium two hours after infection, killed with gentamicin in concentration 1 g/L (Sigma, St. Louis, MO, United States), and rinsed three times with Iscove's medium with 2% human AB serum (Sigma, St. Louis, MO, United States).

Next, culture medium with or without (control) the test compounds at a concentration of 2x MIC was added to independent cultures of the infected macrophages, followed by incubation at 37°C for 48 h under a humidified atmosphere of 10% CO2–90% air. Finally, the macrophages were lysed with 0.1% SDS, and the number of CFUs (colony forming units) was determined as previously described [59].

1.3. Evaluation of InhA inhibition

InhA activity was followed by a colorimetric assay that measured the oxidation of NADH at 340 nm in the presence of 2-trans-octanoyl-CoA in a buffer that contained 30 mM PIPES, pH 7.5, 50 mM NaCl, 0.1 mM EDTA, and 100 nM InhA. This was preincubated for 10 min at room temperature with 0.25 mM NADH and varying concentrations of the compounds with 1% (v/v) DMSO in a 150 μL reaction volume. The reaction was started by the addition of 2-trans-octanoyl-CoA at a final concentration of 1.5 mM. Reactions were followed for 20 min using a plate reader (CLARIOstar, BMG LABTECH) [60].

2. Molecular modeling and Molecular dynamic simulations

The M. tuberculosis InhA protein's structural coordinates were obtained in pdb format from the RCSB PDB [47]. Using AutoDockTools, non-protein moieties, such as water, were eliminated with the exception of NAD, and hydrogen atoms were inserted [61]. Marvin Sketch was used to create the 2D and 3D geometric structures of the 3, 6, and 7 compounds. AutoDock Vina was selected to forecast binding affinities and protein-ligand interactions in the current docking study [62]. The co-crystallized ligand's center (x, y, x; 9.6, 32.2, 60.7) defined the grid box, which was 22.5Åx17.0Åx16.7Å size. The docking results were analyzed and visualized using Discovery Studio Visualizer [63]. To get further insights in regard to the interaction stability of compound **7** within InhA binding site, *i.e.*, protein-ligand stability, molecular dynamics (MD) calculations were carried out on the best docking pose. The input files for MD calculations were generated using CHARMM-GUI solution builder [64,65] using CHARMM force field parameters for InhA. The topologies of the selected ligands were generated using CHARMM General Forcefield through CgenFF server. The CHARMM-GUI solution builder includes five steps. In the first step, the tool reads the coordinates of the protein-ligand complex. The second step involves the solvation of the protein-ligand complex and the determination of the shape and size of the system. Na^+ and Cl^- ions are added in this step to neutralize the system. Periodic Boundary Conditions (PBC) are set in the third step, which is used for the approximation of a large system by using a unit cell, which is then replicated in all directions. The simulation takes place only for the atoms that are present inside the PBC box. Complexes have been neutralized by adding [66] **Na+** ions and have 77 Å, 77 Å, and 77 Å dimensions for the PBC box in x, y, and z, respectively. Bad contacts are removed in this step by running short minimization. The fourth and fifth steps involve the equilibration of the system and production. Equilibration is done in two phases-NVT ensembles and an NPT ensemble to ensure that the system has achieved the desired temperature and pressure. The input files for equilibration and production are then downloaded, and desired changes are made, which include a number of steps of MD run, frequency of saving of trajectories, and calculation of energy, etc. Non-bonded interactions were treated with a 12 ˚A cutoff distance, and the neighbor searching list was buffered with the Verlet cutoff scheme, and the long-range electrostatic interactions were treated with the particle mesh Ewald (PME) method. Prior to production simulation, energy minimization of the system was carried out by using the steepest descent algorithm (5000 steps). The complex was then equilibrated to stabilize

its temperature and pressure by subjecting it to NVT and NPT ensemble and simulating for 125 ps at 300.15 K temperature using 400 kJ mol^{-1} nm^{-2} and 40 kJ mol^{-1} nm^{-2} positional restraints on the backbone and side chains, respectively. Finally, the complex is subjected to a production simulation run for 100 ns in an NPT ensemble at 300.15 K and 1 bar. A Nose-Hoover thermostat was used to maintain the temperature, and similarly, a Parrinello-Rahman barostat was used to maintain the pressure. The LINCS algorithm was used to constrain H-bonds using the inputs provided by CHARMM-GUI. The V-rescale thermostat at 300 K with a coupling constant of 1 ps was used. The trajectories were stored every 2 ps. Simulations of 100 ns in NPT assembly were performed for the production stage. GROMACS 2020.2 [67] software was used to carry out 100ns MD simulation for the complexes. GROMACS utilities were used for the analysis of the MD simulation trajectories. The VMD molecular graphics program [68] was used for trajectory visualization, detailed hydrogen bonding information, and contact frequency analysis. MM/PBSA (Molecular Mechanics/Poisson−Boltzmann Surface Area) calculations were done using g_mmpbsa, a GROMACS tool used to calculate an estimated binding affinity [69].

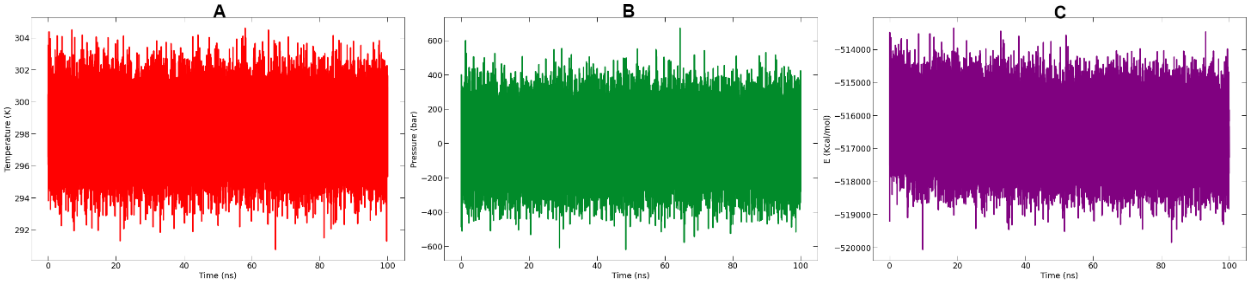

Figure S1. Plot of system parameters **(A)** Temperature, **(B)** pressure and **(C)** potential energy during the 100ns MD simulations

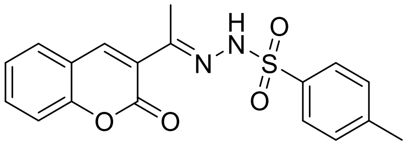

2

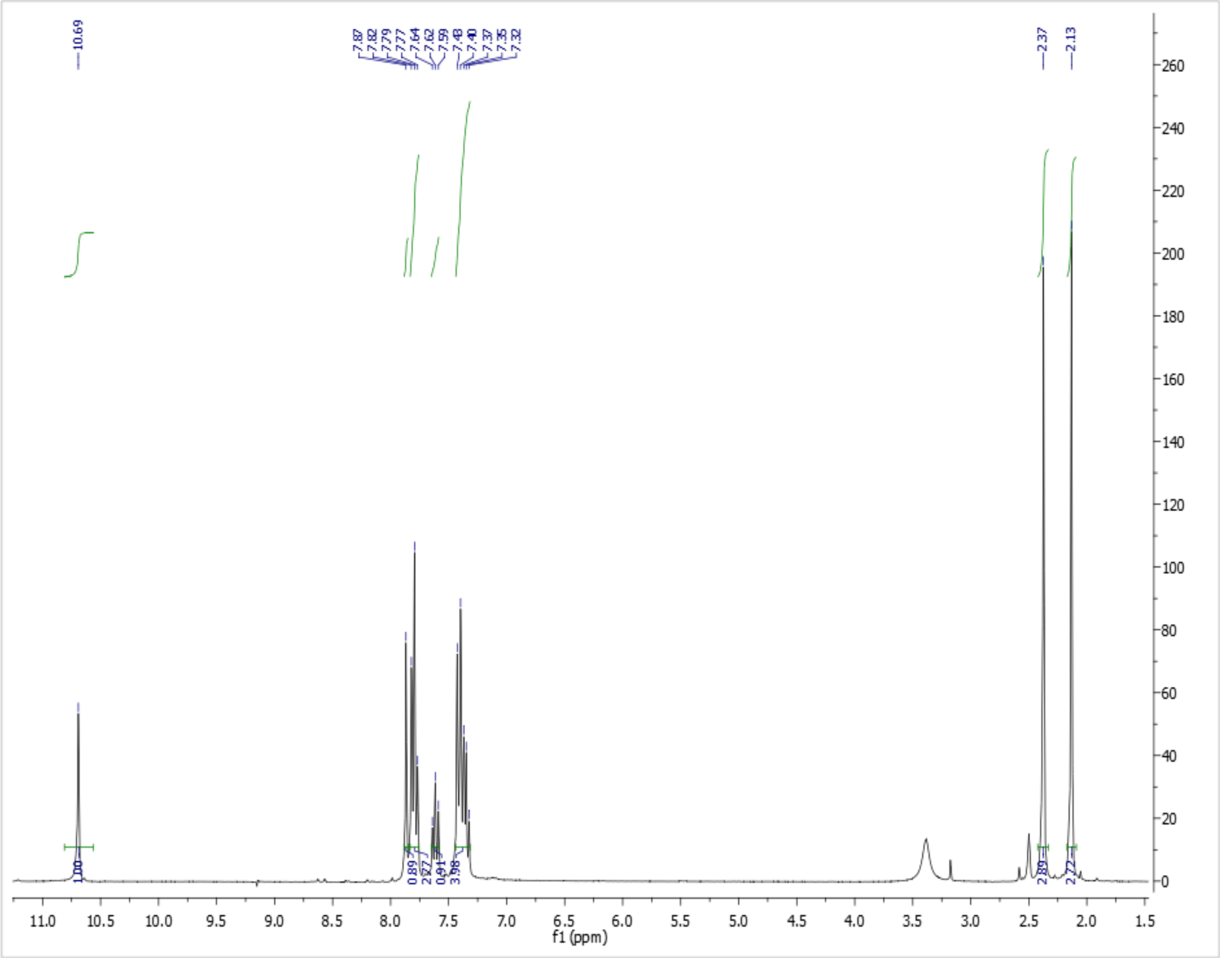
10.69
7.87
7.82
7.79
7.77
7.64
7.62
7.59
7.43
7.40
7.37
7.35
7.32
2.37
2.13
1.00
0.89
2.27
0.91
3.98
2.80
2.72
f1 (ppm)

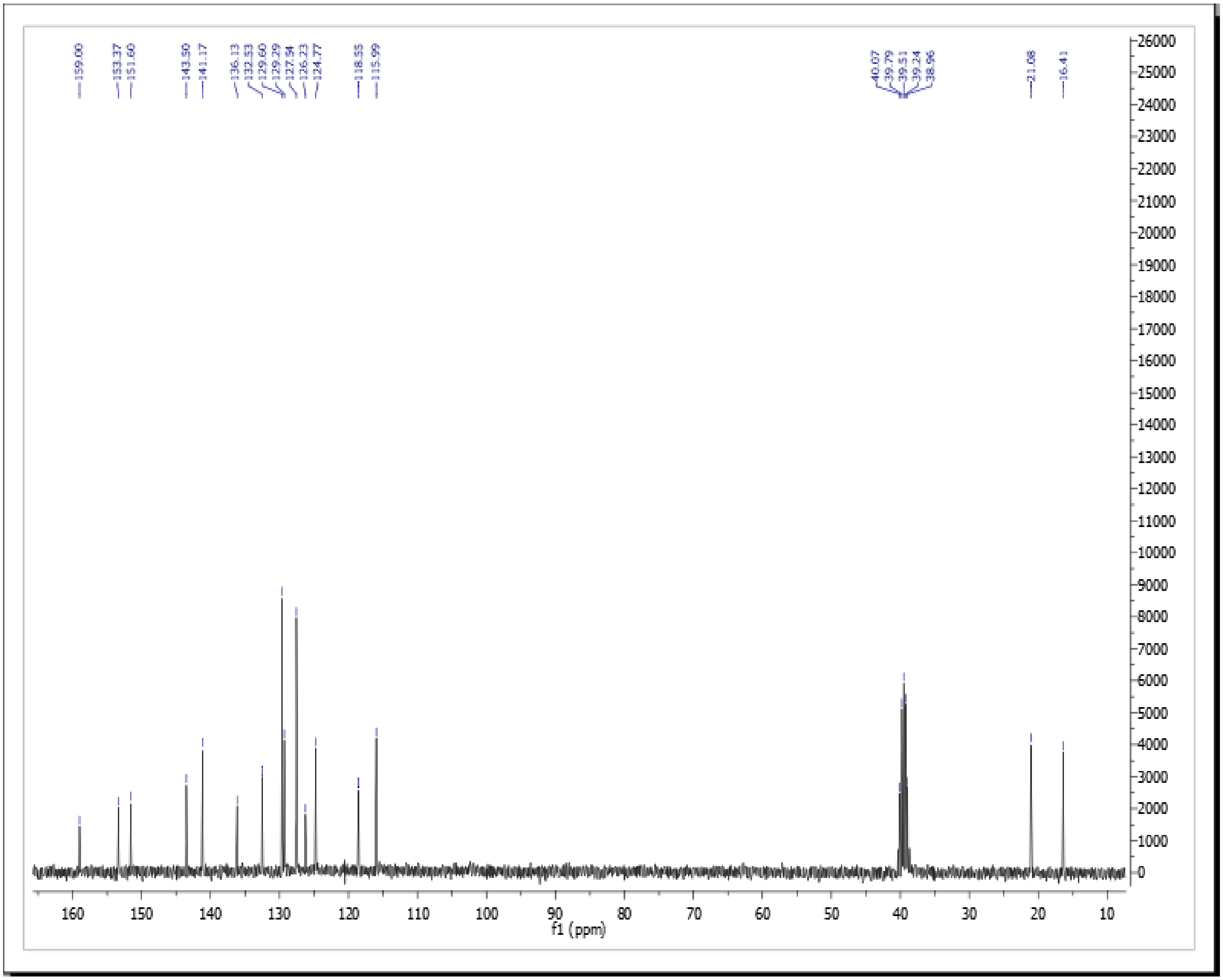
159.00
153.37
151.60
143.50
141.17
136.13
132.53
129.60
129.29
127.54
126.23
124.77
118.55
115.99
40.07
39.79
39.51
39.24
38.96
21.08
16.41
f1 (ppm)

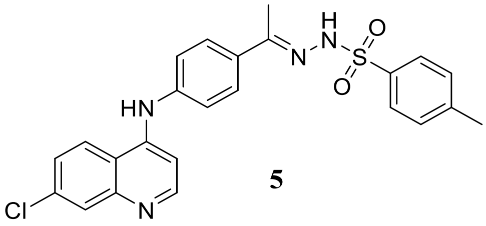

HN
N
H
N
N
S
O
O
Cl

5

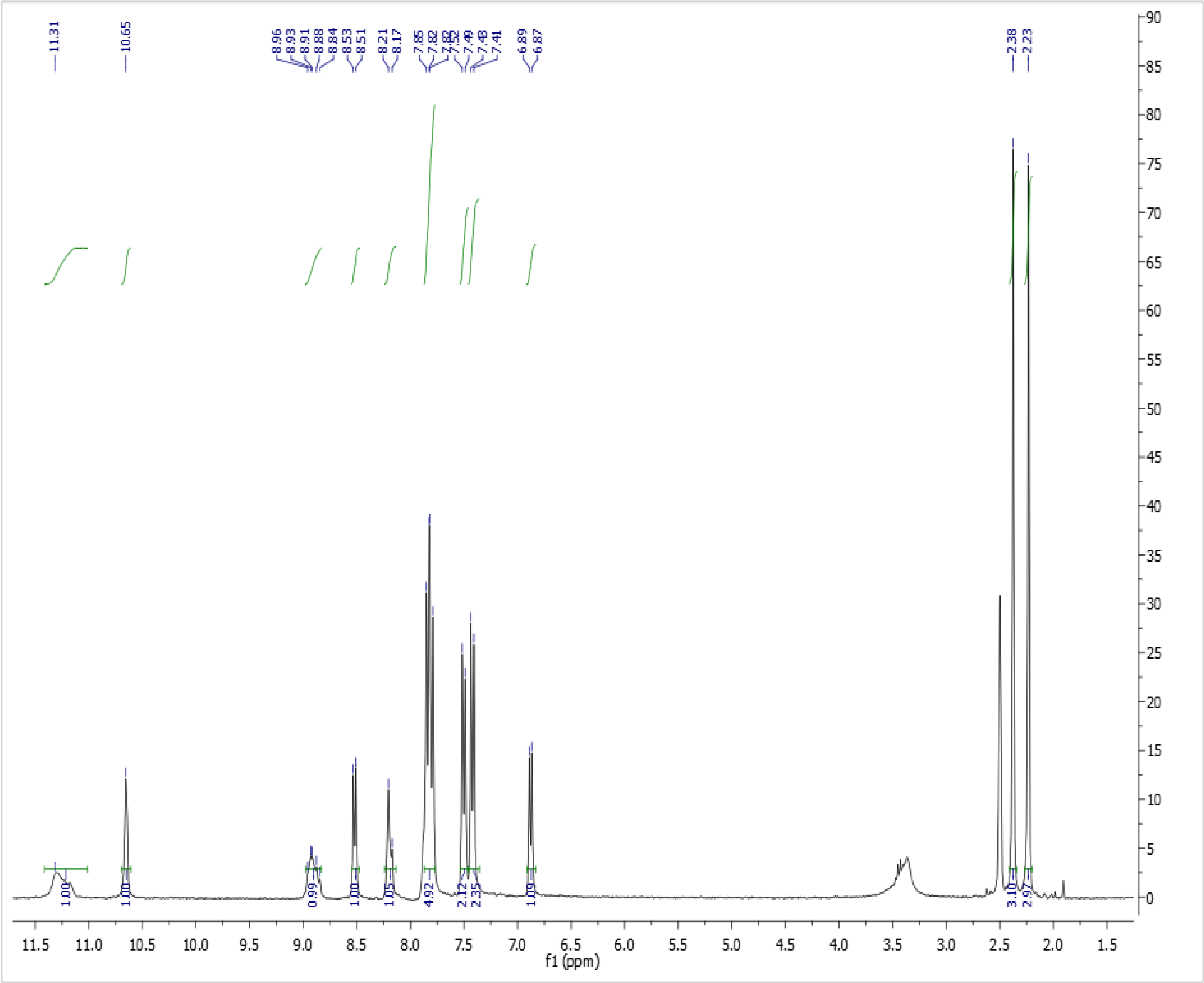

11.31
10.65
8.96
8.93
8.91
8.88
8.84
8.53
8.51
8.21
8.17
7.85
7.82
7.49
7.48
7.41
6.89
6.87
2.38
2.23
1.00
1.00
0.99
1.00
1.05
4.92
2.12
2.35
1.09
3.10
2.97
f1 (ppm)

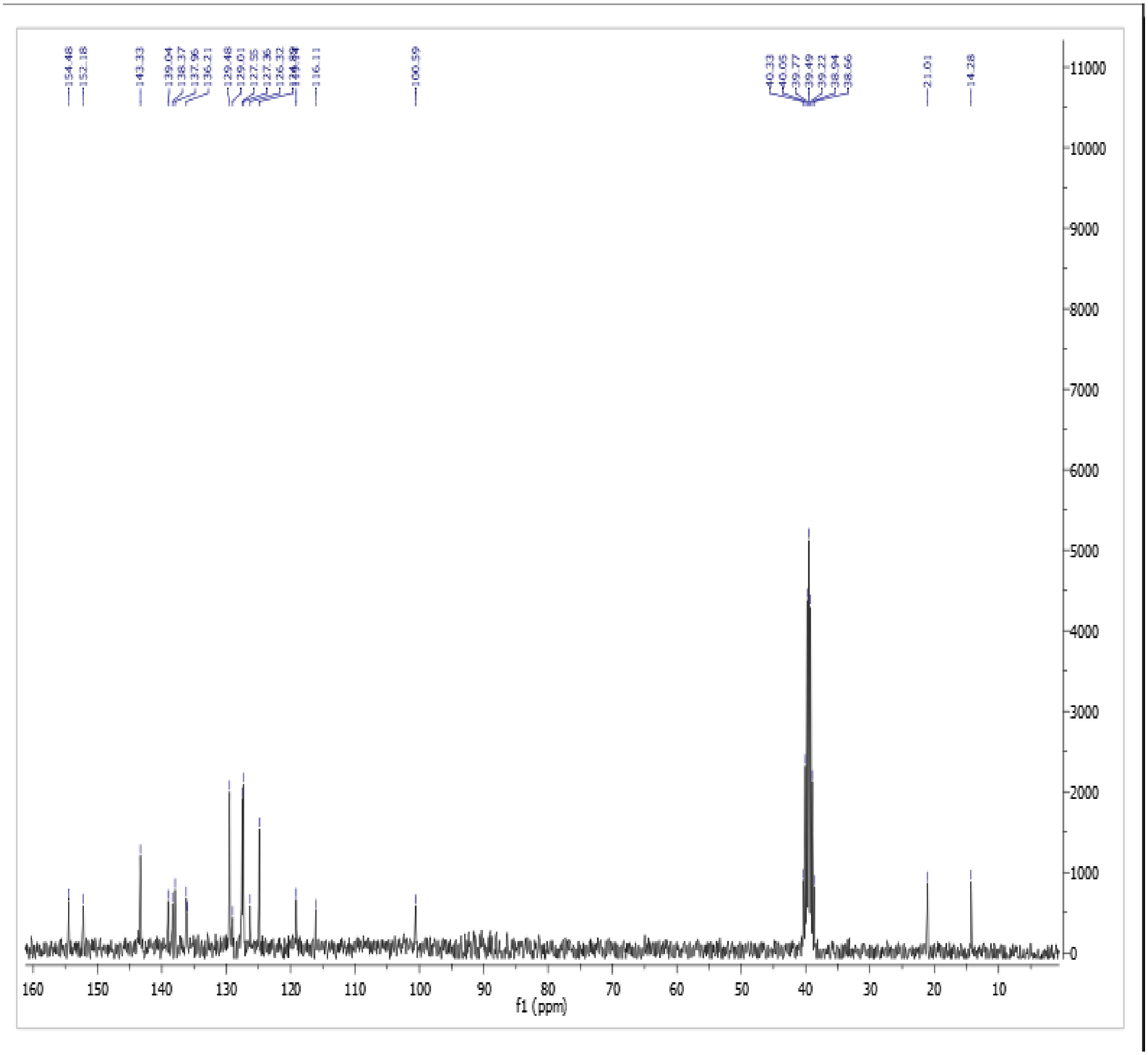

154.48
152.18
143.33
139.04
138.37
137.96
136.21
129.48
129.01
127.55
127.36
126.32
116.11
100.59
40.33
40.05
39.77
39.49
39.22
38.94
38.66
21.01
14.28
f1 (ppm)
